# Significance of α-Myosin Heavy Chain (*MYH6*) Variants in Hypoplastic Left Heart Syndrome and Related Cardiovascular Diseases

**DOI:** 10.3390/jcdd9050144

**Published:** 2022-05-03

**Authors:** Melissa Anfinson, Robert H. Fitts, John W. Lough, Jeanne M. James, Pippa M. Simpson, Stephanie S. Handler, Michael E. Mitchell, Aoy Tomita-Mitchell

**Affiliations:** 1 Department of Cell Biology, Neurobiology, and Anatomy, Medical College of Wisconsin, Milwaukee, WI 53226, USA; manfinson@mcw.edu (M.A.); jlough@mcw.edu (J.W.L.); 2 Herma Heart Institute, Children’s Wisconsin, Milwaukee, WI 53226, USA; shandler@chw.org (S.S.H.); mmitchell@chw.org (M.E.M.); 3 Department of Biological Sciences, Marquette University, Milwaukee, WI 53233, USA; robert.fitts@marquette.edu; 4 Department of Pediatrics, Children’s Mercy, Kansas City, MO 64108, USA; jmjames@cmh.edu; 5 Department of Pediatrics, Division of Quantitative Health Sciences, Medical College of Wisconsin, Milwaukee, WI 53226, USA; psimpson@mcw.edu; 6 Department of Pediatrics, Division of Pediatric Cardiology, Children’s Wisconsin, Milwaukee, WI 53226, USA; 7 Department of Surgery, Division of Congenital Heart Surgery, Children’s Wisconsin, Milwaukee, WI 53226, USA

**Keywords:** hypoplastic left heart syndrome, cardiac myosin heavy chain, congenital heart disease, rare variant analysis

## Abstract

Hypoplastic left heart syndrome (HLHS) is a severe congenital heart disease (CHD) with complex genetic inheritance. HLHS segregates with other left ventricular outflow tract (LVOT) malformations in families, and can present as either an isolated phenotype or as a feature of a larger genetic disorder. The multifactorial etiology of HLHS makes it difficult to interpret the clinical significance of genetic variants. Specific genes have been implicated in HLHS, including rare, predicted damaging *MYH6* variants that are present in >10% of HLHS patients, and which have been shown to be associated with decreased transplant-free survival in our previous studies. *MYH6* (α-myosin heavy chain, α-MHC) variants have been reported in HLHS and numerous other CHDs, including LVOT malformations, and may provide a genetic link to these disorders. In this paper, we outline the *MYH6* variants that have been identified, discuss how bioinformatic and functional studies can inform clinical decision making, and highlight the importance of genetic testing in HLHS.

## 1. Background

Hypoplastic left heart syndrome (HLHS) is a complex form of congenital heart disease (CHD) characterized by hypoplasia of the left ventricle and proximal aorta, as well as stenosis or atresia of the mitral and/or aortic valves [1]. Although significant evidence exists for a genetic basis of HLHS [2,3], its inheritance is multifactorial, complicating the identification of specific genetic risk factors. HLHS occurs in the context of larger chromosomal abnormalities (e.g., Turner [4] and Jacobson syndromes [5]), but also exists as an isolated disorder [6,7]. Additionally, there is an increased incidence of bicuspid aortic valve (BAV), atrial septal defect (ASD), and other left ventricular outflow tract (LVOT) malformations in family members of HLHS patients. Genes implicated in non-syndromic HLHS include *MYH6* [8,9], *NOTCH1* [10], *NKX2.5* [11], *ERBB4* [12], *HAND1* [13], and *GJA1* [14].

We previously identified 19 distinct, rare, predicted damaging *MYH6* variants in a cohort of 190 unrelated HLHS subjects, comprising >10% of the cohort [8]. These findings are consistent with previous studies of mutations in the zebrafish *MYH6* homologue, *amhc/myh6*, wherein loss-of-function mutations, along with *amhc* morpholino knockdown, disrupted atrial sarcomere assembly, impaired atrial contractility, and resulted in atrial dilation in zebrafish embryos. Mutant embryos also exhibited ventricular wall thickening and a narrowed ventricular lumen, mimicking the HLHS phenotype [15]. The additional characterization of knockdown embryos revealed that abnormal ventricular morphology was not due to differences in cardiomyocyte number, but rather due to differences in the size and shape of ventricular cardiomyocytes in *myh6* mutants, compared to wild-type [16]. Similarly, developing *myh6−/− Xenopus tropicalis* hearts lacked cardiac contractility, which was accompanied by atrial and ventricular dilation, and impaired outflow tract development [17]. Although murine models are widely used to study CHD, their cardiac chamber-specific expression of MHC is opposite that of humans, making them unsuitable for modeling *MYH6*-associated disease.

*MYH6* encodes for the alpha isoform of the cardiac myosin heavy chain (α-MHC), which is expressed throughout the myocardium during early cardiac development. As development proceeds, *MYH6* expression decreases in the ventricles and is replaced with *MYH7* (β-MHC) throughout gestation; α-MHC is the dominant atrial isoform postnatally [18,19,20]. Genetic variants in both *MYH6* and *MYH7* have been linked to numerous human cardiac pathologies, including hereditary cardiomyopathies, arrhythmias, as well as CHD. While *MYH7* variants have been characterized more extensively, the specific mechanisms underlying *MYH6* variants are less understood. In this paper, we outline the *MYH6* variants that have been reported in HLHS and other CHDs, discuss the benefits and limitations of biostatistical methods for interpreting variants, and emphasize the importance of mechanistic studies designed to improve personalized treatment strategies.

## 2. Genetic Studies

### 2.1. Known MYH6 Variant Disease Associations

A possible pathogenic role for *MYH6* was first reported more than 30 years ago in a family with hypertrophic cardiomyopathy (HCM). A disease locus, “FHC-1”, was initially identified in all affected but no unaffected members of this family [21]. Investigators later determined that the pathogenicity of the FHC-1 locus was due to a hybrid MYH6/MYH7 gene, in which intron 26 of MYH6 was joined to intron 27 of MYH7 [22]. In the decades since, improvements in next-generation sequencing technology and awareness of the significance of MYH6 in CHD has led to discovery of additional disease-associated MYH6 variants through both family-based and CHD cohort studies. Many groups have reported MYH6 variants in association with septal defects [23,24,25,26,27] (most commonly ASD) [28,29,30,31], as well as in various types of arrhythmias and sudden cardiac death [32,33,34,35,36,37,38,39,40,41]. MYH6 variants are also associated with all types of cardiomyopathy, including HCM [42,43,44,45,46], dilated cardiomyopathy (DCM) [41,47,48,49,50,51,52], peripartum cardiomyopathy (PPCM) [53], arrhythmogenic right ventricular cardiomyopathy (ARVC) [54], and left ventricular non-compaction (LVNC) [55,56]. MYH6 variants have also been identified in patients with Shone complex [27], mitral valve prolapse (MVP) [57], coarctation of the aorta (CoA) [58], and, of relevance to this review, HLHS [23,25,26,59]. Remarkably, HLHS and other LVOT malformations are associated with 49% of all reported MYH6 variants. A list of published MYH6 coding sequence variants and associated CHDs is shown in Table 1.

### 2.2. Clinical Interpretation of Genetic Studies

These genetic studies have been highly informative, but limitations remain in connecting the knowledge of *MYH6* variants to clinical phenotypes. In cohort studies, the designation of a variant as disease-causing relies on a combination of allele frequency and bioinformatic tools of pathogenicity, which are subject to change as new information is learned. There is not a single consensus on what constitutes a “rare” variant, or what is considered damaging when using a continuous variant scoring system, such as Combined Annotation Dependent Depletion (CADD). It is even more challenging to interpret the significance of variants when there are conflicting assessments between computational predictions and variant frequency, or between multiple predictive methods, for example, the *MYH6* variants Q277H, M436V, I512T, V606I, D629N, R860H, A936V, R1151Q, A1298V, D1316E, and R1398Q (Table 1), which have predicted opposite effects when compared using the popular tools SIFT and Polyphen2.

Family-based studies remain a useful way of identifying pathogenic variants, as the segregation of a gene variant within multiple affected family members provides strong evidence that a variant is disease-causing. Familial studies are also directly informative for clinical practice when determining whether family members of an affected individual should be screened for the variant, and if carriers should be surveilled for future disease development. These considerations are particularly important in HLHS due to its high heritability and segregation with other LVOT malformations in family members [66,67], many of which are also associated with *MYH6* variants.

### 2.3. Impact of MYH6 Variants on Outcomes in HLHS

**Figure 1:**
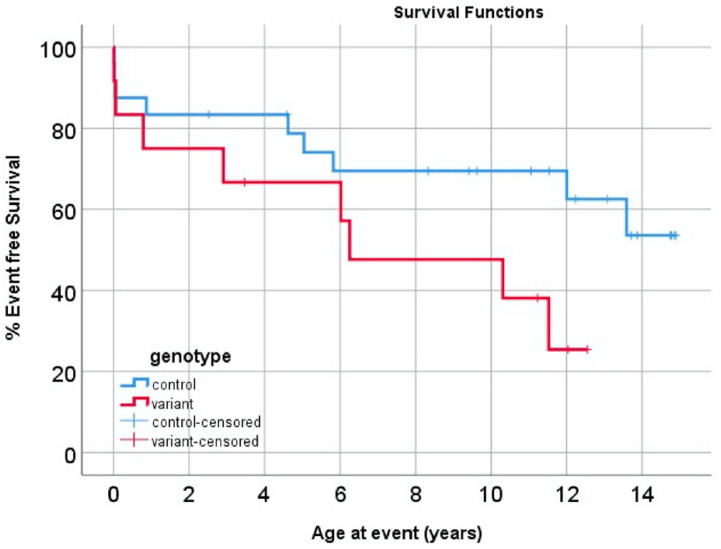
Event-free survival analysis comparing 36 HLHS patients. A total of 12 patients had rare, predicting damaging *MYH6* variants. *p*-value = 0.074, log-rank test.

Our group has examined outcomes of patients with HLHS stratified by presence of an *MYH6* variant. Specifically, we compared a composite endpoint of cardiac arrest, need for mechanical circulatory support, and heart transplant or death between 12 HLHS patients with *MYH6* and 24 HLHS patients without *MYH6* variants. In this cohort, each *MYH6* variant carrier was matched to two controls based on anatomical subtype (i.e., aortic and mitral valve anatomy), stage I surgical shunt type, age/era, and sex when possible. Patients with chromosomal abnormalities and those carrying *MYH7* variants were excluded from this analysis. The difference in reaching the composite endpoint at 15 years between *MYH6* variant and control groups did not reach statistical significance in this small study (Figure 1). However, there is certainly a trend towards improved short-term event-free survival in the control group. Control group outcomes appear better than previously reported transplant-free survival of HLHS patients during follow-up of the single ventricle reconstruction (SVR) randomized trial cohort, which examined differences in transplant-free survival and interventions based on stage I surgical shunt type [68]. These findings warrant further investigation with a larger sample size and emphasize the importance of genetic testing for all HLHS patients to identify variants that may impact survival even more so than surgical shunt type.

## 3. Mechanisms of *MYH6* Variant Pathology

### 3.1. Importance of Mechanistic Studies

Understanding the specific mechanism of *MYH6* variant pathogenicity would be especially relevant to clinical decision making, considering the availability of the drugs omecamtiv mecarbil and mavacamten, which act specifically on the cardiac myosin heavy chains (MHC) to improve systolic and diastolic function, respectively. In phase III clinical trials, both drugs showed efficacy in the treatment of heart failure in adults [69,70,71,72], irrespective of genetic background; omecamtiv mecarbil was FDA-approved for use earlier this year, and FDA approval of mavacamten is pending. In HLHS patients with a known pathogenic *MYH6* variant, the cardiac specificity of omecamtiv mecarbil and mavacamten may offer a way to prevent disease progression. This treatment may be particularly important in variant carriers, given our previous report that HLHS patients with *MYH6* variants have decreased cardiac transplant-free survival compared to HLHS patients without *MYH6* variants [8]. However, choosing to use a cardiac MHC-specific activator vs. inhibitor requires the understanding of whether a specific variant will cause systolic or diastolic dysfunction. This highlights the importance of mechanistic studies designed to understand phenotypes at the cellular and tissue levels. Relative to the large body of literature assessing *MYH7* variants, few studies have sought to understand *MYH6* variant pathology at the molecular level; the findings from these studies are summarized in Table 2.

### 3.2. In Vitro Mechanistic Studies

Most of the mechanistic studies of *MYH6* variants have utilized in vitro methods. The first variant to be functionally assessed was *MYH6*-I820N; this ASD-associated variant is located within the regulatory light chain (RLC) binding region of α-MHC and was found in cellular studies to decrease the binding affinity of α-MHC for RLC [31]. However, RLC binding is thought to modulate MHC activation [76] and it is unclear how a disruption in this process could lead to an ASD. Similarly, the SSS-associated *MYH6*-E933del variant enhanced binding to myosin-binding protein C (MyBP-C), an effect consistent with the location of E933del within the MyBP-C binding region of the α-MHC protein [39]. HL-1 mouse atrial cardiomyocytes transfected with human *MYH6*-E933del also exhibited a slower electric propagation velocity compared to human *MYH6*-WT [39], and neonatal rat ventricular cardiomyocytes (NRVCMs) transfected with either the E933del or the R721W [35] variant exhibited disrupted sarcomere structure and the perinuclear aggregation of α-MHC [39]. Together, these findings led the authors to suggest that structural changes within the atrial cardiomyocytes surrounding the sinus node leads to node dysfunction and conduction defects. However, given the role of MyBP-C in modulating contractile strength [77], it is unclear how changes in the α-MHC/MyBP-C interaction are linked to the identified conduction deficits.

Many other *MYH6* variants have shown similar changes in sarcomere structure. Cultured cardiomyocytes expressing the A230P [26], A1366D [26], E526K [64], R1822_E1823dup [73], and HLHS-associated R443P [8] variants exhibited decreased sarcomere organization in variant-carrying cells, while H252Q actually increased myofibril striations [26]. Interestingly, the *MYH6*-V700M variant did not appear to impact sarcomere organization, despite its rare frequency (<0.001%) and being predicted as “likely damaging” by CADD, SIFT, and PolyPhen2. Our lab also assessed sarcomere organization in cardiac tissue from HLHS patients and found that atrial sarcomeres were disrupted with the R443P, K849del, and E1503V variants, while the ventricular sarcomere structure remained intact [75], consistent with α-MHC being the predominant atrial MHC isoform postnatally. Other groups have also evaluated cardiac tissue from *MYH6* variant carriers and reported fibrosis in the conduction system [36], ventricular walls [36,44,54], and ventricular septum [44,54]. Given the predominance of β-MHC in the postnatal ventricles, it is possible that ventricular fibrosis is, at the cellular level, a downstream response to atrial cardiomyocyte dysfunction caused by *MYH6* variants in the patients studied.

Some of the most informative in vitro studies are those that examine contractility at the cellular level. NRVCMs expressing human *MYH6*-A1004S shortened at a slower rate, and consequently shortened less overall, when compared to NRVCMs expressing human *MYH6*-WT. In the same study, NRVCMs expressing human *MYH6*-P830L showed no difference in shortening rate, compared to *MYH6*-WT [74]. This finding is somewhat unexpected, given that A1004S is located on the MHC backbone and is predicted by both SIFT and PolyPhen2 to be non-damaging (Table 1). The *MYH6*-A1004S variant is also found at a frequency of 1.1% in the general population, which is more common than CHD. Meanwhile, *MYH6*-P830L is a novel variant and located near the RLC binding region and thus one would predict greater changes in function with P830L than A1004S. Our lab also found that the *MYH6*-R443P variant decreased the shortening rate, relaxation rate, extent of shortening, percent shortening, and calcium transient amplitude at the single CM level in patient-specific induced pluripotent stem-cell-derived cardiomyocytes (iPSC-CMs), without affecting action potentials. These *MYH6*-R443P iPSC-CMs also demonstrated sarcomere disorganization and the upregulation of *MYH7*, recapitulating the phenotype found in atrial tissue from an HLHS patient carrying the R443P variant [8,75].

### 3.3. In Vivo Mechanistic Studies

To date, zebrafish embryos are the only animal model that has been used to study human *MYH6* variants. Specifically, researchers evaluated the ability of the human *MYH6*-E933del and *MYH6*-R1252Q variants, which are associated with cardiac conduction disease, to rescue cardiac impairments resulting from *myh6* knockdown. In both sets of experiments, the authors reported bradycardia in knockdown embryos at 48 hpf −137.7 ± 2.2 bpm in *myh6*−/− vs. 150.2 ± 1.6 in uninjected [39], and 144 ± 16 bpm in *myh6*−/− vs. 153 ± 13 in uninjected [67]. While human *MYH6*-WT and *MYH6*-R1252Q increased heart rate in knockdowns, human *MYH6*-E933del failed to rescue this phenotype.

### 3.4. Structural Considerations

**Figure 2:**
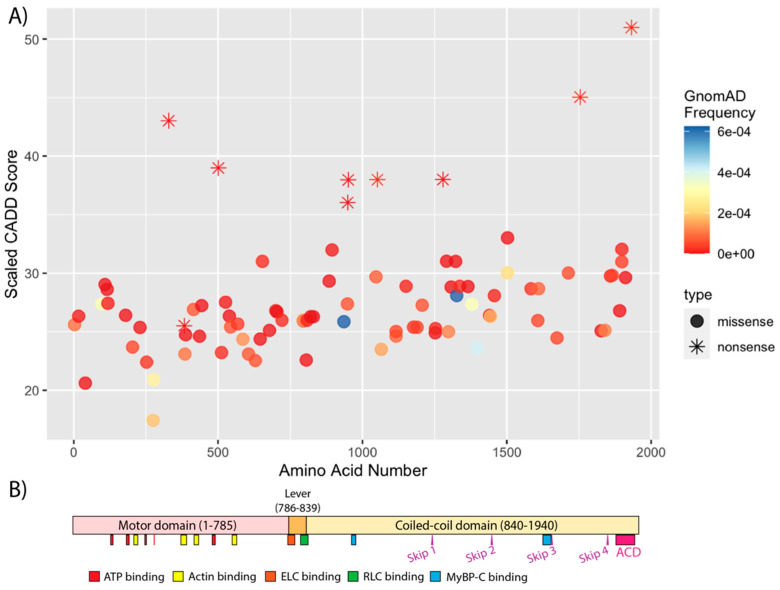
(**A**) Location of rare, predicted damaging *MYH6* coding sequence variants by residue. Variants were considered rare if allele frequency was <1 × 10^−3^ in both the Genome Aggregation Database (gnomAD) Genomes dataset v3.1.2 and the Allele Frequency Aggregator (ALFA) dataset (release version 20201027095038). Single nucleotide variants were considered predicted damaging if the scaled Combined Annotation Dependent Depletion (CADD) score was >22.0 (GRCh37, v1.6) [60], or if the variant was predicted “damaging” or “probably damaging” by SIFT [61] and PolyPhen2 [62]. Deletions are not shown as CADD scores cannot be calculated. (**B**) Schematic of α—MHC domains. ELC, essential light chain; RLC, regulatory light chain; MyBP—C, myosin binding protein C; ACD, assembly competent domain.

The structure of human α-MHC has not been solved, thus most hypotheses regarding the effect of *MYH6* variants on the α-MHC structure are based on comparison to the solved structures of β-MHC. Mutational clustering analysis using population-level data found that pathologic variants in *MYH7* cluster in certain regions [78], which has been used to inform ACMG/AMP variant classification framework [79]. At present, no such “mutational hotspots” have been identified in *MYH6* (Figure 2); however, similar patterns could emerge as new pathological variants are discovered. Investigating structure-function relationships in *MYH7* has been successful in elucidating a mechanism for HCM that results from variants in a surface region of β-MHC referred to as the “myosin mesa” [80,81]. However, the interpretation of such studies may be complicated by the finding that the same *MYH7* variant can cause clinically opposite phenotypes (i.e., both HCM and DCM), depending on the person [82].

Some research groups have begun employing advanced in silico methods to model the effect of specific *MYH6* variants. Molecular dynamics simulations predicted that the *MYH6* variants E1207K and T1379M alter the helicity and flexibility of the tail domain, which would likely impact the rigidity and movement of the thick filament as a whole [9]. Similarly, simulations found that *MYH6*-R1822_E1823dup likely increases the strength of the dimerized α-MHC tail domain, decreasing its flexibility [73]. However, this information does not explain either the clinical or cellular phenotypes associated with these variants.

## 4. Conclusions

HLHS is a complex and genetically heterogenous disease, and the origins of HLHS are likely multigenic. Evidence suggests *MYH6* variants are etiologic in a significant percentage of HLHS. Many of the studies cited in this paper identified additional candidate variants that may be contributing to cardiac disease development, including some patients carrying compound heterozygous *MYH6* variants. New bioinformatic tools, such as Oligogenic Resource for Variant AnaLysis (ORVAL) [83], are designed to identify candidate pathogenic combinations of variants and are likely to be useful in elucidating the multigenic origins of HLHS and related disorders. The presence of additional genetic variants and environmental influences may explain some of the discrepancies between bioinformatic predictions of *MYH6* variant pathogenicity and the reported cellular and clinical phenotypes. In any event, *MYH6* variants will remain an important genetic risk factor for HLHS, having prognostic significance irrespective of other factors.

Our published work, [8,75] and many of the studies discussed in this paper, supports our hypothesis that atrial dysfunction due to sarcomere disorganization impairs atrial contractility during cardiac development leading to HLHS. These changes in atrial cardiomyocytes would likely impair atrial contractility in single ventricle patients postnatally, leading to heart failure over time. We anticipate that future longitudinal analyses will allow us to better understand the impact of *MYH6* variants on long-term cardiac function in HLHS.

## Figures and Tables

**Table 1:** Published *MYH6* coding sequence variants. Minor allele frequencies were obtained from the Genome Aggregation Database (gnomAD) Genomes dataset v3.1.2 and the Allele Frequency Aggregator (ALFA) dataset (release version 20201027095038). Computational methods used to predict deleterious nature of variants include scaled Combined Annotation Dependent Depletion (CADD) score (GRCh37, v1.6) [60], SIFT [61], and PolyPhen2 [62]. Not all computational methods were able to calculate potential pathogenicity scores for truncating variants or deletions. *Abbreviations*: HLHS, hypoplastic left heart syndrome; ASD, atrial septal defect; CHD, unspecified congenital heart disease; DCM, dilated cardiomyopathy; HCM, hypertrophic cardiomyopathy; TA, tricuspid atresia; LVH, left ventricular hypertrophy; TGA, transposition of the great arteries; PFO, patent foramen ovale; MVP, mitral valve prolapse; PPCM, peripartum cardiomyopathy; SSS, sick sinus syndrome; CoA, coarctation of the aorta; WPW, Wolff–Parkinson–White syndrome; AVSD, atrioventricular septal defect; ARVC, arrhythmogenic right ventricular cardiomyopathy; AF, atrial fibrillation; LVNC, left ventricular noncompaction; SDK, septal dyskinesia; SAR, subaortic ridge; AS, aortic stenosis; DIVC, dilated inferior vena cava.

Variant	Associated Phenotype	Scaled CADD Score	SIFT Prediction	PolyPhen2 Prediction	GnomAD Genomes Allele Arequency	ALFA Allele Frequency
D3N	HLHS [63]	25.6	damaging	probably damaging	9.20 × 10^−5^	4.00 × 10^−5^
R17H	ASD [30]	26.3	damaging	probably damaging	6.57 × 10^−6^	not reported
P40R	CHD [23]	20.6	damaging	probably damaging	not reported	not reported
E98K	Shone complex [27], HLHS [63], CHD [26]	27.4	damaging	probably damaging	3.15 × 10^−4^	6.12 × 10^−4^
R108C	HLHS [63]	29	damaging	probably damaging	not reported	not reported
Y115N	HLHS [8]	28.6	damaging	probably damaging	not reported	not reported
S118L	HLHS [63]	27.4	damaging	possibly damaging	6.60 × 10^−6^	not reported
S180Y	DCM [52]	26.4	damaging	probably damaging	not reported	not reported
R204H	HCM [43]	23.7	tolerated	probably damaging	4.60 × 10^−5^	2.20 × 10^−5^
D208N	AVSD [24]	22.3	tolerated	benign	4.15 × 10^−3^	6.38 × 10^−3^
A230P	TA, LVH [26]	25.4	damaging	probably damaging	not reported	not reported
H252Q	TGA, PFO [26]	22.4	damaging	probably damaging	2.63 × 10^−5^	6.00 × 10^−5^
I275N	DCM [48,49]	17.4	damaging	probably damaging	1.84 × 10^−4^	3.63 × 10^−4^
Q277H	HLHS [8,63], CHD [23]	20.9	damaging	benign	2.76 × 10^−4^	4.04 × 10^−4^
E329stop	ASD [29]	43	damaging	N/A	not reported	not reported
D383N	HLHS [8]	25.5	damaging	probably damaging	not reported	not reported
S385L	HLHS [8]	23.1	tolerated	benign	9.87 × 10^−5^	not reported
L388F	Shone complex [27]	24.7	damaging	probably damaging	not reported	not reported
G415R	MVP [57]	26.9	damaging	probably damaging	6.57 × 10^−5^	7.00 × 10^−5^
M436V	HLHS [8]	24.6	tolerated	probably damaging	not reported	not reported
R443P	HLHS [8]	27.2	damaging	probably damaging	not reported	3.00 × 10^−5^
E501stop	TA [26]	39	damaging	N/A	not reported	not reported
I512T	Shone complex [26]	23.2	damaging	benign	1.97 × 10^−5^	3.00 × 10^−5^
E526K	ASD [64]	27.5	damaging	possibly damaging	6.57 × 10^−6^	not reported
C539R	ASD [30]	26.3	damaging	possibly damaging	not reported	not reported
K543R	ASD [30]	25.4	damaging	possibly damaging	5.93 × 10^−5^	1.20 × 10^−4^
R568C	PPCM [53], DCM [48]	25.7	damaging	probably damaging	3.29 × 10^−5^	9.00 × 10^−5^
G585S	Shone complex [27]	24.4	damaging	possibly damaging	1.31 × 10^−4^	1.91 × 10^−4^
D588A	HLHS [8,9,63]	22.6	tolerated	benign	1.40 × 10^−3^	2.51 × 10^−3^
V606I	HLHS [63]	23.1	damaging	benign	6.57 × 10^−5^	not reported
D629N	HCM [44]	22.5	tolerated	possibly damaging	5.91 × 10^−5^	7.00 × 10^−5^
F646L	HLHS [63]	24.4	damaging	possibly damaging	not reported	not reported
R654W	Arrhythmia [33]	31	damaging	probably damaging	1.97 × 10^−5^	4.00 × 10^−5^
N678S	ASD, TA [25]	25.1	damaging	probably damaging	not reported	7.00 × 10^−5^
V700M	PFO [26,65]	26.8	damaging	probably damaging	6.57 × 10^−6^	not reported
I704N	HLHS [9]	26.7	damaging	probably damaging	not reported	not reported
R721W	SSS [35], CoA [58]	26	damaging	probably damaging	1.91 × 10^−5^	not reported
R795Q	HCM [42]	25.9	damaging	probably damaging	not reported	1.00 × 10^−4^
R795W	HLHS [8]	26	damaging	probably damaging	1.38 × 10^−4^	2.54 × 10^−4^
I806T	DCM [41]	22.6	tolerated	benign	not reported	not reported
R809C	HCM [43]	26	damaging	probably damaging	1.31 × 10^−5^	8.00 × 10^−5^
I820N	ASD [31]	26.3	damaging		not reported	not reported
P830L	DCM [47,50]	26.3	damaging	probably damaging	not reported	not reported
K849del	HLHS [8]	N/A	N/A	N/A	not reported	not reported
T864M	HLHS [63]	20.7	tolerated	benign	7.89 × 10^−5^	1.40 × 10^−4^
E885K	WPW [32]	29.3	damaging	probably damaging	not reported	not reported
A895V	CHD [26]	32	damaging	possibly damaging	6.57 × 10^−6^	not reported
E933del	SSS [39]	N/A	N/A	N/A	not reported	not reported
A936V	HLHS [8], AVSD [24]	25.9	tolerated	possibly damaging	6.24 × 10^−4^	2.16 × 10^−4^
E948K	MVP [57], HLHS [63]	27.4	damaging	probably damaging	5.91 × 10^−5^	3.00 × 10^−5^
C949stop	ARVC [54]	36	damaging	N/A	not reported	not reported
E951stop	ASD [29]	38	damaging	N/A	not reported	not reported
A964S	HLHS [8]	26.3	tolerated		1.37 × 10^−3^	1.76 × 10^−4^
A1004S	ASD [30], DCM [47,48,50]	23.6	tolerated	benign	1.10 × 10^−3^	1.03 × 10^−3^
R1047C	DCM [51]	29.7	damaging	probably damaging	7.23 × 10^−5^	2.00 × 10^−5^
R1052stop	MVP [57]	38	damaging	N/A	1.97 × 10^−5^	3.00 × 10^−5^
Q1065H	HCM [47]	23.5	damaging	probably damaging	1.51 × 10^−4^	1.10 × 10^−4^
I1068T	Shone complex [27]	21.9	tolerated	benign	not reported	not reported
R1116S	ASD [26]	24.6	damaging	probably damaging	7.90 × 10^−5^	7.00 × 10^−5^
R1116C	HCM [66]	26	damaging	probably damaging	not reported	not reported
R1116H	SSS [67]	25	damaging	probably damaging	4.60 × 10^−5^	7.00 × 10^−5^
R1151Q	HLHS [8]	28.9	tolerated	probably damaging	1.32 × 10^−5^	not reported
R1177W	DCM [48]	25.4	damaging	probably damaging	2.64 × 10^−5^	4.00 × 10^−5^
T1190I	HLHS [63]	25.4	damaging	probably damaging	3.95 × 10^−5^	not reported
E1207K	HLHS [9]	27.3	damaging	probably damaging	5.93 × 10^−5^	5.00 × 10^−5^
R1252Q	SSS [67]	24.9	damaging		not reported	not reported
T1253M	DCM [51]	25.3	tolerated	probably damaging	1.31 × 10^−5^	not reported
R1279stop	ASD [28]	38	damaging	N/A	not reported	not reported
R1291P	HLHS [63]	31	damaging	probably damaging	not reported	not reported
A1298V	HLHS [8]	25	tolerated	possibly damaging	1.12 × 10^−4^	2.00 × 10^−4^
K1307M	AF [34]	28.8	damaging	probably damaging	not reported	not reported
D1316E	SCD [37]	19.7	tolerated	possibly damaging	1.31 × 10^−5^	3.00 × 10^−5^
E1323V	AF [34]	31	damaging	probably damaging	not reported	not reported
A1327V	Shone complex [27]	28.1	damaging	probably damaging	6.21 × 10^−4^	1.70 × 10^−4^
S1337L	LVNC [55]	28.9	damaging	probably damaging	1.98 × 10^−5^	4.00 × 10^−5^
A1366D	SDK, SAR, PFO, AS [26]	28.9	damaging	probably damaging	not reported	not reported
T1379M	HLHS [8,9], MVP [57], CHD [26]	27.3	damaging	probably damaging	3.22 × 10^−4^	5.87 × 10^−4^
R1398Q	HLHS [63], CHD [23]	23.6	damaging	benign	4.14 × 10^−4^	5.26 × 10^−4^
A1440P	DCM [48]	26.4	tolerated	possibly damaging	not reported	not reported
A1443D	ASD [26,65], HLHS [8,63]	26.3	damaging		1.71 × 10^−4^	1.30 × 10^−4^
E1457K	DCM [47,50]	28.1	damaging	probably damaging	1.31 × 10^−5^	not reported
R1502Q	DCM [48,49]	30	damaging	probably damaging	2.17 × 10^−4^	2.61 × 10^−4^
E1503V	HLHS [8]	33	damaging	probably damaging	not reported	not reported
E1584K	HLHS [8]	28.7	damaging	probably damaging	2.63 × 10^−5^	not reported
R1608C	CHD [23]	26	damaging	probably damaging	4.60 × 10^−5^	8.00 × 10^−5^
R1610C	Shone complex [27]	28.7	damaging	probably damaging	7.89 × 10^−5^	6.00 × 10^−5^
A1674T	CHD [41]	24.5	tolerated	benign	4.60 × 10^−5^	3.00× 10^−5^
E1713K	HLHS [63]	30	damaging	probably damaging	5.26 × 10^−5^	not reported
E1754stop	HLHS [8]	45	damaging	N/A	1.97 × 10^−5^	not reported
E1827D	HLHS [63]	25.1	damaging	probably damaging	not reported	not reported
K1840R	HLHS [8] HCM [43]	25.1	tolerated	probably damaging	1.12 × 10^−4^	2.50 × 10^−4^
D1859N	HLHS [63]	29.8	damaging	probably damaging	1.94 × 10^−5^	not reported
R1865Q	DIVC, ASD, VSD [26]	29.8	damaging	probably damaging	4.06 × 10^−5^	3.00 × 10^−5^
A1891T	Shone complex [27]	26.8	damaging	probably damaging	not reported	not reported
R1899C	DCM [48]	32	damaging	probably damaging	1.31 × 10^−5^	not reported
R1899H	Shone complex [27]	31	damaging	probably damaging	3.94 × 10^−5^	3.00 × 10^−5^
R1911P	HLHS [63]	29.6	damaging	probably damaging	not reported	not reported
K1932stop	Shone complex [27]	51	damaging	N/A	1.97 × 10^−5^	not reported

**Table 2:** Functional studies evaluating the role of *MYH6* variants in cardiac disease.

Model System	*MYH6* Variant	Disease Association	Cellular Phenotype	Reference
Purified recombinant protein	I820N	ASD	↓ binding affinity of α-MHC to RLC	[31]
HeLa cells	E933del	SSS	↓ binding affinity of α-MHC to MyBP-C	[39]
Myofibrils differentiated from C2C12 myoblasts	A230P	TA, ASD, LVH	↓ myofibrillar organization	[26]
	A1366D	AS, SDK, SAR, PFO	↓ myofibrillar organization	[26]
	H252Q	TGA, PFO	↑ myofibril striations	[26]
	V700M	PFO	No effect on myofibrillar organization	[26]
	E526K	ASD	↓ myofibrillar organization No impact on actin-activated ATPase activity	[64]
	R1822_ E1823dup	ASD	↓ myofibrillar organization No impact on cell viability ↑ apoptosis	[73]
Rat or mouse ventricular myocytes expressing human *MYH6*	R721W	SSS, CoA	↓ myofibril striations MHC aggregation	[39]
	P830L	DCM	No effect on peak contraction, shortening velocity, Ca homeostasis, or relaxation time	[74]
	E933del	SSS	↓ propagation velocity ↓ myofibril striations MHC aggregation	[39]
	A1004S	ASD, DCM	↓ peak contraction (with and without isoproterenol stimulation) ↓ shortening velocity No change in Ca homeostasis or relaxation time	[74]
Induced pluripotent stem-cell-derived cardiomyocytes (iPSC-CMs)	R443P	HLHS	↓ cardiomyogenic differentiation Disorganized sarcomeres ↓ shortening and relaxation rates ↓ extent of shortening, % shortening ↓ amplitude of Ca transient No effect on action potential ↑ expression of sarcomere genes, including *MYH7* (β-MHC)	[8,75]
Patient cardiac tissue	R443P	HLHS	Atrial sarcomere disarray	[8]
	N598fs	ACM	LV and septal fibrosis	[54]
	D629N	HCM	RV and septal myocyte disarray	[44]
	A822T	SCD	LV and conduction system fibrosis	[36]
	K849del	HLHS	Atrial sarcomere disarray (no effect on ventricular sarcomere organization)	[8,75]
	E1503V	HLHS	Atrial sarcomere disarray (no effect on ventricular sarcomere organization)	[75]
	S385L & M436V	HLHS	No effect on atrial or ventricular sarcomeres	[75]
Zebrafish	E933del	SSS	↓ heart rate of *MYH6* knockout was rescued by wild-type *MYH6*, but not variant	[39]
	R1252Q	SSS	↓ heart rate, stroke volume, cardiac output, fractional area change of *MYH6* knockout were rescued by wild-type *MYH6*, but not variant	[67]

## Data Availability

Restrictions apply to the availability of these data. Data is restricted as it contains protected patient information.
